# “Sticky Bone” Preparation Device: A Pilot Study on the Release of Cytokines and Growth Factors

**DOI:** 10.3390/ma15041474

**Published:** 2022-02-16

**Authors:** Ezio Gheno, Gutemberg Gomes Alves, Roberto Ghiretti, Rafael Coutinho Mello-Machado, Antonio Signore, Emanuelle Stellet Lourenço, Paulo Emílio Correa Leite, Carlos Fernando de Almeida Barros Mourão, Dong-Seok Sohn, Mônica Diuana Calasans-Maia

**Affiliations:** 1 Post-Graduation Program in Dentistry, Fluminense Federal University, Niteroi 24220-140, RJ, Brazil; eziogheno@gmail.com (E.G.); rafaelcoutinhodemello@yahoo.com.br (R.C.M.-M.); emanuelle_stellet@yahoo.com.br (E.S.L.); 2 Surgical Sciences and Integrated Diagnostics Department, University of Genoa, 16132 Genoa, Italy; dr.signore@icloud.com; 3 Cell and Molecular Biology Department, Institute of Biology, Fluminense Federal University, Niteroi 24220-000, RJ, Brazil; gutopepe@yahoo.com.br; 4 Clinical Research Unit of the Antonio Pedro Hospital, Fluminense Federal University, Niteroi 24220-000, RJ, Brazil; mouraocf@gmail.com; 5 Maxillofacial Surgeon, Private Practitioner, 46047 Porto, Italy; roberto.ghiretti@libero.it; 6 Implant Dentistry Department, Universidade Iguaçu, Nova Iguaçu 26260-045, RJ, Brazil; 7 Therapeutic Dentistry Department, Institute of Dentistry, I.M. Sechenov First Moscow State Medical University, 119435 Moscow, Russia; 8 Post-Graduation Program in Sciences and Biotechnology, Fluminense Federal University, Niteroi 24220-000, RJ, Brazil; leitepec@gmail.com; 9 Department of Dentistry and Oral and Maxillofacial Surgery, Catholic University Medical Center of Daegu, Daegu 705-718, Korea; dssohn@cu.ac.kr; 10 Department of Oral Surgery, Dentistry School, Fluminense Federal University, Niteroi 24020-140, RJ, Brazil

**Keywords:** PRP, PRF, growth factors, bone regeneration, bone graft, sticky bone

## Abstract

Sticky bone, a growth factor-enriched bone graft matrix, is a promising autologous material for bone tissue regeneration. However, its production is strongly dependent on manual handling steps. In this sense, a new device was developed to simplify the confection of the sticky bone, named Sticky Bone Preparation Device (SBPD^®^). The purpose of this pilot study was to investigate the suitability of the SBPD^®^ to prepare biomaterials for bone regeneration with autologous platelet concentrates. The SBPD^®^ allows the blending of particulate samples from synthetic, xenograft, or autogenous bone with autologous platelet concentrates, making it easy to use and avoiding the need of further manipulations for the combination of the materials. The protocol for the preparation of sticky bone samples using the SBPD^®^ is described, and the resulting product is compared with hand-mixed SB preparations regarding in vitro parameters such as cell content and the ability to release growth factors and cytokines relevant to tissue regeneration. The entrapped cell content was estimated, and the ability to release biological mediators was assessed after 7 days of incubation in culture medium. Both preparations increased the leukocyte and platelet concentrations compared to whole-blood samples (*p* < 0.05), without significant differences between SB and SBPD^®^. SBPD^®^ samples released several growth factors, including VEGF, FGFb, and PDGF, at concentrations physiologically equivalent to those released by SB preparations. Therefore, the use of SBPD^®^ results in a similar product to the standard protocol, but with more straightforward and shorter preparation times and less manipulation. These preliminary results suggest this device as a suitable alternative for combining bone substitute materials with platelet concentrates for bone tissue regeneration.

## 1. Introduction

Platelet concentrates have been used for bone and soft tissue healing in alveolar ridge augmentation, periodontal surgery, socket preservation, implant surgery, endodontic regeneration, sinus augmentation, bisphosphonate-related osteonecrosis of the jaw (BRONJ), osteoradionecrosis, closure of oroantral communication (OAC), and oral ulcers [1,2].

As the use of platelet concentrates in dentistry is continually evolving, many modifications of their original production protocols have been proposed, ranging from novel obtention methods to the proposal of new devices to improve and facilitate handling during surgical procedures [3], platelet-rich fibrin (PRF) protocols [2,3] and from the original platelet-rich plasma (PRP) [4] to advanced centrifugation steps [5,6], combinations with albumin gels, and injectable PRF preparations [7,8,9]. 

With advancements in surgical techniques, some authors have also proposed the association of platelet concentrates with bone graft materials such as synthetic bone, xenografts and allografts. A new concept of fabricating a growth factor-enriched bone graft matrix, also known as “sticky bone” (SB), using autologous fibrin-rich blocks with concentrated growth factors (CGF), was first demonstrated by Sohn and colleagues by 2011 [10] and later by Mourão et al. [11] with liquid/injectable platelet-rich fibrin matrices (i-PRF). By 2015, Sohn et al. provided a complete description of the optimized protocol for the production of the sticky bone composed of a CGF membrane and growth factor-enriched bone graft matrix, along with a series of clinical cases supporting its use, pointing to a stabilization of the bone graft in a defect that contributes to minimal bone loss during the healing period [12]. 

These autologous biomaterials present a significant release of cytokines and autologous growth factors contributing to healing and tissue recovery [13,14], even though there is still no consensus on their actual influence on the formation of bone tissue [15]. Nevertheless, the ease of handling, the consequent reduction in surgical time, and the agglutination of the biomaterial for bone regeneration are already factors contributing to the surgical grafting procedure [16]. 

However, the available protocols for sticky bone preparation involve the manual mixing of the bone substitute and the blood derivative, which may cause disruption of the clot, loss of biomaterial, and impaired reproducibility of the resulting composite. A novel, device has been developed and patented to simplify the confection of the sticky bone, named Sticky Bone Preparation Device (SBPD^®^). The device produces combinations of particulate bone substitute materials and platelet concentrates without manual handling during the mixing steps. The present work aimed to compare the resulting biomaterial with hand-mixed SB preparations regarding in vitro parameters such as cell content and the ability to release growth factors and cytokines relevant to tissue regeneration. 

## 2. Materials and Methods

This in vitro pilot study was reported following the criteria of the Science in Risk Assessment and Policy (SciRAP) guideline (www.scirap.org (accessed on 25 November 2021)), which are based on the Guidance Document on Good In Vitro Method Practices (GIVIMP) [17]. A checklist indicating the conformity with the guidelines is provided in Supplementary Table S1. 

### 2.1. Ethical Considerations

Blood samples were collected with the informed consent form signed by a healthy volunteer donor, without comorbidities such as blood diseases and diabetes, and who was not taking any medication. Blood tests were performed to ensure normality. The present research is part of a project approved by the Universidade Federal Fluminense Research Ethics Committee (CAAE number 12126919.7.0000.5243) and was performed following the ethical standards of the ethics research committee and the 1964 Helsinki declaration.

### 2.2. Device Description

**Figure 1:**
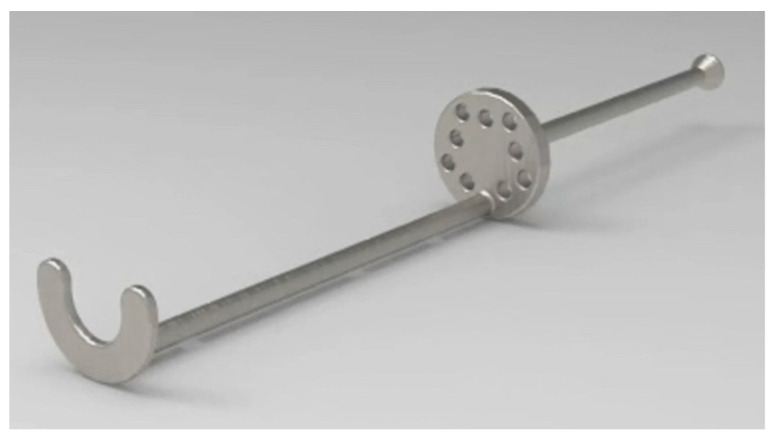
The Sticky Bone Preparation Device (SBPD^®^).

The device (Figure 1) was produced with grade 4 titanium, consisting of a 2 mm stem with a length of 87 mm. It has a conical appendix of 2.5 mm at its lower terminal with a base of 4 mm. In the middle portion, 38 mm from the bottom, there is a filter with a diameter of 12 mm and a thickness of 2 mm. The filter has nine holes with a diameter of 1.5 mm to allow the flow of the corpuscular portion of the blood and retain the particulate bone substitute. At the top of the SBPD^®^, a 1 mm thick half-moon allows the removal of the device along with the particulate biomaterial mixed with platelet aggregate by the end of the centrifugation steps. The device patent is registered at the European Patent Office (No. 102020000022009).

The use of the device is primarily intended for the association of platelet concentrates, produced through different centrifugation protocols, with small bone particulates of autologous, homologous, heterologous, or synthetic origin.

### 2.3. Sticky Bone Preparation

**Figure 2:**
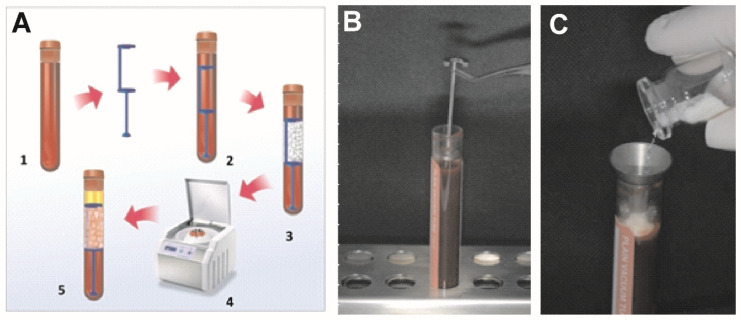
(**A**) The sequence of steps of sticky bone preparation with the SBPD. The process starts with the peripheral blood collection (Step 1), followed by the placement of the device (Step 2), the addition of particulate biomaterial (Step 3), and centrifugation according to the platelet aggregate protocol chosen (Step 4). It ends with the removal of the SBPD^®^ with the complete sticky bone preparation (Step 5). (**B**) Detailed view of the insertion of the SBPD into the tube. (**C**) Detailed view of the sterile metallic funnel and the particulate bone substitute being inserted.

Figure 2 shows a schematic design for the “sticky bone” produced by SBPD^®^. Venous blood samples (6 mL) were collected from the donor with the kit BD Vacutainer^®^ Safety-Lok™ in a red cap tube (10 mL) without any additives (Figure 2A). At the end of the collection, the test tube cap was removed, and the filter SBPD^®^ was inserted into the tube (Figure 2B). To produce the SB, 500 mg of bovine xenograft (Bonefill^®^ Bioinnovation Biomedical, São Paulo, Brazil) was employed as the particulate bone substitute and added through a small sterile metallic funnel. The bone substitute remained in the upper portion of the tube (Figure 2C). The cap was replaced to seal the tube again. The test tube was placed in the centrifuge, making sure to place another one equipped with SBPD in the opposite region to counterbalance its weight, and the centrifugation was then carried out according to the Concentrated Growth Factors (CGF) protocol [18], using a Medifuge device (Silfradent, Italy) (Figure 2A) to stratify the blood according to the weight of its component. The bone particulate remained in the portion above the filter. The centrifugation steps included a 30 s acceleration and sequential centrifugations at 2700 rpm/4 min, 2400 rpm/4 min, 2700 rpm/4 min, and 3000 rpm/3 min, followed by a 36 s deceleration. The Medifuge device applied all these variations automatically, and the whole process took about 13 min. 

After centrifugation, the xenograft remained in the device portion above the filter, along with a gelatinous product composed of the produced CGF. The SBPD^®^ was pulled together with these particulates using the extraction half-moon. The particulate bone substitute mixed with the CGF matrix was then separated from the SBPD^®^, ready to use (Figure 2C).

**Figure 3:**
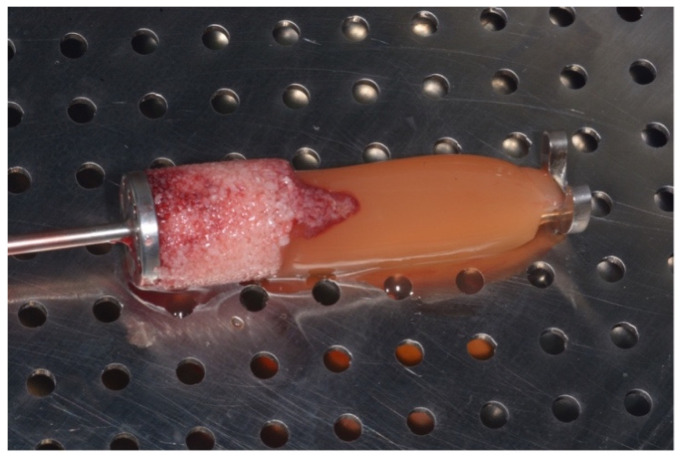
The particulate bone substitute mixed with the CGF (“sticky bone”) prepared with the SBPD^®^ after centrifugation.

After centrifugation, a stratification of the product was observed, with a dense and gelatinous upper layer, which was the CGF fibrin portion. The lower portion was the so-called “sticky bone”, a mix of fibrine bone particulate and growth factors (Figure 3). The particulate bone substitute was firmly agglomerated in the fibrin clot produced by the blood centrifugation. It contained a high concentration of all those products useful to trigger the regenerative process and neo-angiogenesis.

To determine if the use of the SBPD affected the biological properties of the resulting material, samples were also produced, for comparison, without the use of the preparation device. These were produced from samples of the same donor, according to the protocol proposed by Temmerman et al. [16], adapted to the production of CGF. Briefly, CGF membranes were produced from 6 mL blood samples, collected in red cap tubes (10 mL) without any additives, and centrifuged with the same sequential steps described above on a Medifuge device (Silfradent, Italy). The CGF was collected and mixed with 500 mg of the bovine xenograft (Bioinnovation Biomedical, São Paulo, Brazil) using a sterile metal cube. After around 5 min of polymerization, it was possible to obtain the sticky bone (SB). 

### 2.4. Estimation of Cellularity

The estimation of red blood cells, leukocytes, and platelets was performed according to the subtraction method. The sticky bone preparations (*n* = 3 per experimental group) were pressed on a PRF box (Biohorizons, Birmingham, AL, USA). Four metal stoppers of 1 mm were included on each side of the PRF box to standardize the handling pressure, membrane thickness, and the quantity of released exudate. The fibrin clot exudates were collected and placed into tubes containing EDTA (Greiner Bio-One, São Paulo, Brazil). During processing, the supernatant serum fractions (PPP) and the bottom semi-coagulated thrombus with red blood cells (RBCs) (1 cm below the area referring to the buffy coat) were also collected and pooled with the exudate in the EDTA-containing tubes. The tubes were homogenized for the complete disintegration of clots. A further sample was collected on the EDTA-containing tube, representing the “whole-blood” sample. To quantify RBCs, leukocytes, platelets, and platelets, these samples were evaluated with a Counter 19 CP Hematology Analyzer (Wiener Lab, Rosario, Argentina). The cell content on the sticky bone was estimated using the subtraction method, where

*Sticky bone cell count* = “*whole blood*” − *post-processing blood fractions* (*Exudate* + *PPP* + *RBC*).

### 2.5. Evaluation of the Release of Growth Factors

To verify the release of cytokines and growth factors into the biological media from the “Sticky Bone” preparations, an in vitro assessment was performed according to a previously described methodology [17]. The sticky bone and whole-blood samples (*n* = 3 per experimental group) were prepared as described and cultured for 7 days after their preparation in six-well culture plates (TPP, Merck KGaA Darmstadt, Germany) in the presence of 4 mL of DMEM medium (Dulbecco’s Modified Eagle’s Medium, GIBCO, Waltham, MA, USA), without antibiotics, in a humidified atmosphere at 37 °C and 5% CO_2_. Aliquots of the conditioned medium were collected at the end of the incubation and stored in a freezer at −80 °C.

The concentration of cytokines and growth factors was detected in these samples through a multiparametric immunoassay based on XMap-labeled magnetic microbeads (LuminexCorp, Chicago, IL, USA). A commercial kit (27-plex panel, Biorad Inc., Hercules, CA, USA) was employed; it was capable of quantifying IL-1β, IL-10, IL-12 (p70), IL-13, IL-15, IL-10, IL-10, IL- IL-17, CCL11, FGF-b, CSF3, CSF2, IFN-γ, CXCL10, CCL2, CCL3, CCL-4, PDGF, CCL5, TNFα, and VEGF levels. Quantification of the magnetic beads was performed with a BioPlex MAGPIX system (Biorad Inc., Hercules, CA, USA). Results were analyzed using the Xponent v. 3.0 software (Luminexcorp, Chicago, IL, USA).

### 2.6. Statistical Analysis

Results were calculated as means and standard errors of the mean (SEMs). The evaluators were unaware of the sample identities during analysis. After a D’Agostino–Pearson normality test, the results from the cell counts were compared through a nonparametric Kruskal–Wallis test with a Dunn post hoc test. The results from the cytokine and growth factor evaluation were compared through multiple *t*-tests, always considering an alpha error of 5%, using the software GraphPad Prism 7.0 (GraphPad, San Diego, CA, USA).

## 3. Results

**Figure 4:**
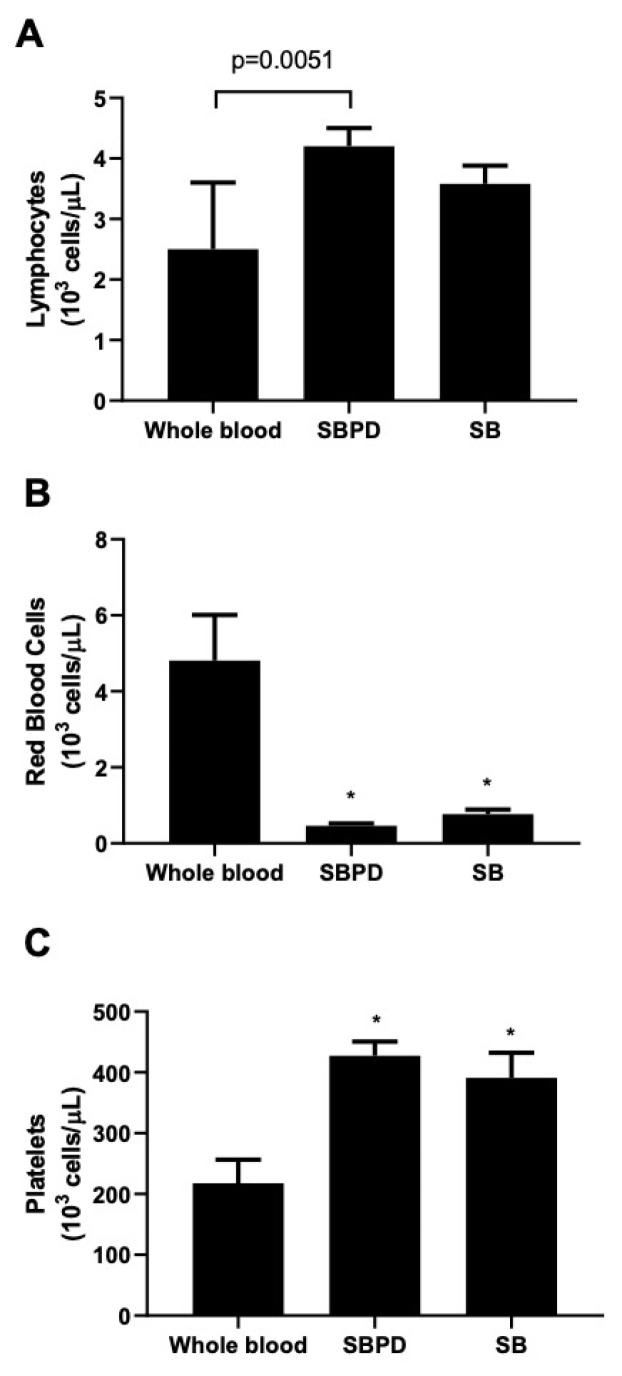
Cell content of the hand-mixed (SB) and SBPD^®^ sticky bone preparations, as compared to whole-blood samples, represented by the mean ± SD of lymphocytes (**A**), red blood cells (**B**), and platelets (**C**). An asterisk (*) indicates a significant difference from the whole-blood group (*p* < 0.05). Results indicate the mean ± SD of three biological replicates with three technical replicates.

Regarding the cell content after the processing of blood samples with the SBPD, Figure 4 shows that the platelet concentrations obtained were within the normal laboratory reference range (170–400 × 10^3^/µL). By estimating the total amount of cells (considering the cell concentrations the recovered volume of eluates), more than 90% of the platelets and leukocytes were retained on the sticky bone matrix (data not shown). The SBPD preparation achieved almost a twofold increase in their concentration (*p* < 0.05), with a much lower content of red blood cells as compared to the whole blood samples, both for the SB and SBPD preparations. There was no statistical difference between SB and the samples prepared with SBPD^®^ for any cell count.

The sticky bone preparation’s ability to release growth factors and cytokines was assessed through a multiparametric immunoassay after 7 days of incubation on culture medium. The preparation did not present evidence of bacterial contamination even after 7 days of culture without antibiotics. From the 27 analytes assessed, 14 presented detectable levels, as described in Table 1. These included the growth factors PDGF, VEGF, bFGF, and GM-CSF, the proinflammatory interleukins (IL)-6, IL-15, and IL-5, the anti-inflammatory mediators IL-10 and IL-1RA, and the chemokines RANTES, MCP-1, MIP-1b, eotaxin, and IP-10. The levels of most analytes were quite similar between SBPD and SB, but significant differences were found for FGFb, IL-5, MCP-1, and MIP-1b, which were released significantly more by SBPD^®^ samples after 7 days (*p* < 0.05). 

## 4. Discussion

A variety of platelet concentration techniques have been introduced in the surgical field to favor bone tissue regeneration. Platelet-rich plasma (PRP) and platelet rich in growth factors (PRGF) belong to the first generation of platelet concentrates/aggregates requiring anticoagulants with thrombin and/or calcium chloride to induce fibrin clot [3]. Platelet-rich plasma (PRP) may have some benefits in reducing complications such as alveolar osteitis and improving healing of soft tissue of extraction sockets [19]. PRP has revealed better results in periodontal therapy in association with other materials than when it is used alone, suggesting that the specific selection of agents/procedures combined with PRP could be important [20].

The second generation of platelet concentrates/aggregates, initially represented by platelet-rich fibrin (PRF), uses only the patient’s own venous blood without any anticoagulant or other addictive to have the fibrin polymerization [3]. Concentrated growth factor (CGF) was another autologous blood-derived biomaterial, proposed by Sacco in 2006, produced by centrifuging blood samples with specifically centrifugation process using a special centrifuge (Medifuge, Silfradent, Italy). However, while PRF uses a constant spin speed, either by vertical or horizontal centrifugation [21,22], CGF protocols employ variating spin speeds to produce a richer fibrin matrix with growth factors, denser, and larger which facilitates handling during the surgical procedures [10]. Interesting outcomes have been reported for CGF in bone tissue regeneration, such as inducing active new bone formation in the maxillary sinus without postoperative complications [23,24], and contributing to new bone formation in large diaphyseal bone defects by the Masquelet technique [25]. It also has been proposed in modified protocols combining its fibrin matrix with denatured albumin [7], titanium mesh membranes [26], bone morphogenetic protein-2 (BMP-2) [27], or particulate materials such as in sticky bone [10]. 

“Sticky bone” is a type of composite biomaterial that facilitates the bone regeneration process when a particulate bone substitute is used as a scaffold, benefiting from the bioactive properties of an autologous platelet aggregate such as PRF and CGF [10,11,12,16]. Generally, surgeons insert bone graft materials with the use of a saline solution to facilitate transport to the operated site [16], even though these procedures may imply considerable loss of material. Through the combination with platelet concentrates, it is possible to obtain an agglutinated material that allows the graft to be implanted in the host with greater precision and virtually without the loss of particles. In this context, the device presently described facilitates the production of “sticky bone” by the surgeon and/or assistants, as there is no need for more than one handling stage. In contrast, in preparation without the device, it is necessary to produce/collect the enriched plasma, mix with the bone substitute, and wait for agglutination before starting the surgical procedure. In the present protocol, the device allowed for concomitant mixing and centrifugation, resulting in a ready-to-use material for bone regeneration. 

The present results indicate that the use of the device does not affect the content of platelets and leukocytes entrapped on the formed fibrin matrix, as evidenced by the fact that these estimated contents had no significant differences between preparations (with or without the device), as well as a similar cell content increase to that previously described for CGF preparations [28]. These cells are of great relevance for the continuous production of growth factors, which play a key role in the regenerative properties of biomaterials based on platelet aggregates. Previous histological and immunohistochemical analyses of CGF membranes have shown the presence of multiple platelet cell elements forming aggregates trapped among the fibrin network [28]. The analysis of a different sticky bone preparation also identified the homogeneous presence of platelet “islands” (aggregates) surrounded by leukocyte groups [18]. It is important to notice that the estimation of cellularity in the present study relied on the cell counting of the membrane exudates after pressing on a PRF metal box and could be affected by the pressure exerted over the materials. In this sense, this study employed the solution proposed by Dohan Ehrenfest et al. [29], using 1 mm metal stoppers in the PRF box for the standardization of the CGF membrane production. 

The biochemical analysis revealed that both SB and SBPD^®^ preparations can release some of the main growth factors (GFs) involved in bone healing, such as bFGF, VEGF, and PDGF. CGF membranes have already been reported as optimizing the production of GFs [30], with an increased release of mediators of angiogenic inductors such as VEGF and PDGF, as compared to other platelet aggregate preparations. Indeed, high concentrations of both molecules were released by SB and SBPD, without a significant difference between them (*p* > 0.05). On the other hand, fibroblastic growth factor II (FGFb) release was slightly increased with the use of SBPD. This growth factor promotes the cell proliferation of mesenchymal stem cells, fibroblasts, and osteoblasts, impacting the development of tissue neoformation [31]. Nevertheless, it is important to note that such a slight increase (less than 10%) would probably not affect the material’s regenerative properties and would need to be confirmed by further assessments. 

Both materials were also able to release pro- and anti-inflammatory cytokines and chemokines. While the release profile was very similar between SB and SBPD regarding important cytokines involved in bone tissue regeneration such as IL-6 and RANTES, statistical differences were found for IL-5, MCP-1, and MIP-1b (*p* < 0.05). It is not yet clear how IL-5, which experienced a sevenfold increase with the use of SBPD, could affect bone tissue regeneration, even though a study has found evidence of perturbation of bone metabolism with overexpression of this proinflammatory cytokine on a murine in vivo model [32]. Monocyte chemoattractant protein-1 (MCP-1/CCL2), which was also elevated with the use of the sticky bone device, presents a role in bone remodeling [33], as a key mediator of osteoclastogenesis through monocyte and macrophage recruitment, osteoclast formation, and consequent increase in bone resorption. Furthermore, the increased expression of MCP-1, induced by parathyroid hormone (PTH), may be related to increased bone volume on animal tests [34]. Macrophage inflammatory protein 1 beta (MIP-1b), also known as CCL4, may have a complex role in fracture healing that still needs to be investigated, with some reports of differential expression during bone tissue repair [34].

As observed for FGFb, these findings alone are not sufficient to infer that these differences induced using SBPD would be relevant to the outcome of the sticky bone clinical applications. Nevertheless, it is worth noting that the detected levels were comparable to other sticky bone preparations [16], as well as CGF preparations assessed through the same in vitro experimental conditions [7,8], reinforcing the idea that platelet concentrates may also act as immunomodulatory nodules [22].

Among the limitations of the SBPD^®^ is the fact that a single device is restricted to produce materials of the same size for all intended applications. While this is not a limitation that may negatively influence the production of sticky bone, specific uses may demand the production of devices with different dimensions. Furthermore, a more significant amount of red blood cells (RBCs) may be observed in people with a higher hematocrit [35,36]. Nevertheless, in the present study, the quantification of RBCs in the final product did not show significant differences between the preparations with or without the device. 

The present study assessed the use of the SBPD device for the production of sticky bone employing CGF protocols, a combination previously proposed by Sohn et al. [10], which considers the use of altered centrifugation speed to produce larger, denser, and richer fibrin matrices. The present results indicate resulting composite biomaterials with similar biological properties to the original, manual SB protocols [10,18]. However, since this is a pilot study, these preliminary results must still be considered with care, due to the main limitation of the experimental design, i.e., the low number of biological replicates. This study was completely performed with three biological replicates per group (SB and SBPD), all from the same donor, aiming to reduce interferences from individual variability in this initial assessment. On the other hand, this choice strongly limits the external generalizability of the reported results. Considering the means and standard deviations observed for the main growth factors in the piloting, a sample size of seven biological samples would be adequate to achieve a 0.80 power with a 5% alpha error [37]. Therefore, the basis is set for required further assessments, not only to confirm the present preliminary findings with increased statistical power, but also to assess the use of SBPD^®^ with other centrifugation protocols for platelet aggregate production, such as PRF sticky bone, and to determine the outcome of using SBPD through randomized split-mouth clinical trials.

## 5. Conclusions

The Sticky Bone Preparation Device (SBPD^®^) was able to produce composites with similar cell contents to the usual manual handling protocol, as well as the in vitro ability to release growth factors involved in bone healing. These preliminary results suggest it as a potential easy-to-use tool to avoid manual handling steps during the production of combinations of platelet aggregates with bone substitute biomaterials. 

## Figures and Tables

**Table 1:** Content of released growth factors and inflammatory mediators by sticky bone preparations produced with the SBPD^®^, after 7 days of immersion in culture medium. Results indicate the mean ± SD of three biological replicates with three technical replicates. An asterisk indicates a significant difference between groups (*p* < 0.05).

Analyte	Concentration (pg/mL)		*p*-Value
	Regular Sticky Bone	SBPD Preparation	
VEGF	28.16 ± 15.10	30.18 ± 7.21	0.774446
PDGF-BB	7870.11 ± 571.30	8629.05 ± 957.10	0.126216
Basic FGF	9.70 ± 0.10	11.72 ± 0.71 *	0.000042 *
GM-CSF	68.0 ± 20.41	47.66 ± 19.82	0.053537
IL-6	1.91 ± 1.77	1.41 ± 1.50	0.600874
IL-5	0.32 ± 0.61	2.21 ± 0.22 *	0.000029 *
IL-15	0.22 ± 0.0	0.36 ± 0.22	0.150110
IL-1RA	213.25 ± 12.41	271.05 ± 68.0	0.066668
IL-10	0.33 ± 0.09	0.50 ± 0.33	0.251402
MCP-1	17.39 ± 7.53	36.84 ± 10.73	0.004515 *
MIP-1b	56.63 ± 40.08	187.19 ± 36.87 *	0.000163 *
Eotaxin	194.45 ± 22.20	304.07 ± 94.57	0.019087
RANTES	1491.59 ± 920.21	1332.02 ± 491.59	0.714897
IP-10	1208.94 ± 1111.67	2106.48 ± 1006.44	0.054860

## Data Availability

Not applicable.

## Supplementary Materials

The following supporting information can be downloaded at https://www.mdpi.com/article/10.3390/ma15041474/s1: Table S1: SciRAP reporting checklist for in vitro studies.
