# Supplementary material for: “Sticky Bone” Preparation Device: A Pilot Study on the Release of Cytokines and Growth Factors
Source: Materials (Basel). 2022 Feb 16;15(4):1474. doi: 10.3390/ma15041474 (PMC8879971; doi:10.3390/ma15041474)
Supplement: Supplementary file 1 [file materials-15-01474-s001.zip › materials-1505402-supplementary.pdf]

Table S1. SciRAP reporting checklist for in vitro studies.

Category	Items to be described	Location in mansucript
Purpose and aim	Purpose and/or aim of the study.	last paragraph of introduction
Endpoints	Endpoints included in the investigation.	subsection #4 and #5 of Materials and Methods
Test compound	Name, ID and/or CAS-number.	Line 112, Materials and Methods
	Source, i.e. manufacturer and batch/lot number.	Line 113, Materials and Methods
	Purity, including information on contaminants, isomers, etc.	Product data sheet
	Other relevant information, e.g. radiolabelled.	N/A
	Stability and homogeneity of the compound in the vehicle under the conditions of use and storage.	N/A
	Stability in the medium, i.e. sensitivity of the test compond to hydrolysis and/or photolysis.	N/A
	Solubility.	N/A
	Temperature for storage.	subsection #4 of Materials and Methods
Vehicle	Type/characteristics.	No vehycle was used.
	Justification for choice of vehicle if other than ethanol or DMSO.	No vehycle was used.
Ethical statement	Ethical review permissions, licenses and national or institutional guidelines, as relevant.	subsection #1 of Materials and Methods
Test system	Type of system, e.g. cell line, primary cells, tissue, organ, embryo.	subsection #3 of Materials and Methods
	Species and strain (as relevant) of the origin of the cells/tissue/organ.	subsection #3 of Materials and Methods
	Source, i.e. provider of the cells/tissue/organ.	subsection #1 of Materials and Methods
	Metabolic competence.	subsection #1 of Materials and Methods
	The number of cell passages if cell line was used.	N/A
	Composition of the media, including use of serum, antibiotics, etc.	subsection #5 of Materials and Methods
	Incubation temperature, humidity, and CO2 concentration.	subsection #5 of Materials and Methods
	Measures taken for avoiding or screening for contamination by mycoplasma, bacteria, fungi and virus.	subsection #5 of Materials and Methods
Administration of test compound	Cell density or number of cells used.	N/A
	Method for deciding on number of replicates per dose level/concentration or the number of times the experiment was repeated, e.g. power calculations.	last paragraph of Discussion
	Dose levels or concentrations and number of dose groups.	subsection #5 of Materials and Methods
	Rationale for selection of dose levels, e.g. relevant to effects observed in vitro or human exposure levels.	subsection #5 of Materials and Methods
	Information about controls (negative and positive), are they concurrent, historical, matched, etc.	subsection #3 of Materials and Methods
	Duration of treatment e.g. hours, days.	subsection #5 of Materials and Methods
	Frequency of administration, e.g. single, repeated or continuous.	subsection #5 of Materials and Methods
	The number of replicates per dose level/concentration or the number of times the experiment was repeated.	subsections #4 and #5 of Materials and Methods
Methods	Methods should be described in enough detail to allow replication either in the Methods section or in another publication to which a clear reference is made.	Materials and Methods section
	Standards followed, such as good cell culture practice, if relevant.	subsection #1 of Materials and Methods
	Deviations from SOPs.	N/A
	The time points for data collection were stated.	subsections #4 and #5 of Materials and Methods
	Description of the method and how it is relevant to the endpoint being investigated.	Materials and Methods and Discussion
	Data supporting the reliability and sensitivity of the method, i.e. positive control or historical/previously published data or participation in inter-laboratory calibration/validation programs.	Discussion section
	Description of how the effect of the test compound on cytotoxicity was measured.	N/A
	Description of any apparatus used.	Materials and Methods section
	Description of parameters measured.	Materials and Methods and Discussion
	Detection range, limit of detection and limit of quantification.	subsection #5 of Materials and Methods
	Description of blinding of research personnel to the treatment during data collection and analysis, when relevant.	subsections #6 of Materials and Methods
Statistics	Details of statistical methods applied.	subsection #6 of Materials and Methods
	Description that shows that the assumptions of the statistical methods used are fulfilled.	Discussion section, last paragraph
Data	Response data by treatment group.	Results Section
	All data relevant to the endpoints investigated, including statistically significant changes and the appropriate measures of precision/variance should be presented in a transparent manner for all treatment groups, including negative (and positive) controls.	Results Section
Discussion	Description of the dose-response relationships for the measured parameters.	N/A
	How do the results relate to other research within the relevant field, e.g. are the results supported by other research.	Discussion section
	Relevance to humans, e.g. how are the results relevant for human health outcomes or modes of action/key events related to human health outcomes.	Discussion section
Other	List of study personnel, including professional training.	First page
	Contact information for raw data access.	Last page
	Disclosure of any financial conflicts of interest.	Last page
